# Comparative genomic analyses provide insight into the pathogenicity of three *Pseudomonas syringae* pv. *actinidiae* strains from Anhui Province, China

**DOI:** 10.1186/s12864-024-10384-1

**Published:** 2024-05-11

**Authors:** Qian Wang, Yiju Zhang, Rui Chen, Lei Zhang, Min Fu, Lixin Zhang

**Affiliations:** 1 Anhui Province Key Laboratory of Integrated Pest Management on Crops, College of Plant Protection, Anhui Agricultural University, Hefei, China; 2 State Key Laboratory of Vegetable Biobreeding, Tianjin Academy of Agricultural Sciences, Tianjin, China

**Keywords:** Pathogenicity, Complete genome, *Pseudomonas syringae* pv. *actinidiae*, Comparative genomic analyses, Effectors of the type III secretion system

## Abstract

**Background:**

*Pseudomonas syringae *pv.* actinidiae* (Psa) is an important bacterial plant pathogen that causes severe damage to the kiwifruit industry worldwide. Three Psa strains were recently obtained from different kiwifruit orchards in Anhui Province, China. The present study mainly focused on the variations in virulence and genome characteristics of these strains based on the pathogenicity assays and comparative genomic analyses.

**Results:**

Three strains were identified as biovar 3 (Psa3), along with strain QSY6 showing higher virulence than JZY2 and YXH1 in pathogenicity assays. The whole genome assembly revealed that each of the three strains had a circular chromosome and a complete plasmid. The chromosome sizes ranged from 6.5 to 6.6 Mb with a GC content of approximately 58.39 to 58.46%, and a predicted number of protein-coding sequences ranging from 5,884 to 6,019. The three strains clustered tightly with 8 Psa3 reference strains in terms of average nucleotide identity (ANI), whole-genome-based phylogenetic analysis, and pangenome analysis, while they were evolutionarily distinct from other biovars (Psa1 and Psa5). Variations were observed in the repertoire of effectors of the type III secretion system among all 15 strains. Moreover, synteny analysis of the three sequenced strains revealed eight genomic regions containing 308 genes exclusively present in the highly virulent strain QSY6. Further investigation of these genes showed that 16 virulence-related genes highlight several key factors, such as effector delivery systems (type III secretion systems) and adherence (type IV pilus), which might be crucial for the virulence of QSY6.

**Conclusion:**

Three Psa strains were identified and showed variant virulence in kiwifruit plant. Complete genome sequences and comparative genomic analyses further provided a theoretical basis for the potential pathogenic factors responsible for kiwifruit bacterial canker.

**Supplementary Information:**

The online version contains supplementary material available at 10.1186/s12864-024-10384-1.

## Background

Kiwifruit (*Actinidia deliciosa* and *A. chinensis*) is an important economic crop and highly favored by consumers due to its high content of vitamin C and other nutrients [1, 2]. The acreage and production of kiwifruit in China have ranked first in the world in recent years [3]. However, bacterial canker is a destructive disease that causes severe damage to kiwifruit, resulting in substantial economic losses in the kiwifruit industry. Characteristic symptoms of kiwifruit canker include brown leaf spots, discoloration of flower buds, bleeding cankers with white or reddish-brown exudates from branches and trunks, and eventually the death of the plant. The occurrence of the bacterial canker disease in kiwifruit was first confirmed in Japan and spread to a pandemic scale [4, 5]. *Pseudomonas syringae* pv. *actinidiae* (Psa) was first described as a Gram-negative bacterium, rod-shaped with polar flagella, characterized as the causative agent of this disease affecting kiwifruit in Japan [4]. Psa population exhibited different levels of virulence and were divided into five biovars, namely biovars 1, 2, 3, 5, and 6 [6–9]. Different biovars with varying levels of virulence have led to a certain level of complexity regarding the occurrence of this disease [10–12]. Additionally, it has become clear that among these biovars there are remarkable differences in the composition of pathogenicity-related genes, such as genes encoding phytotoxins, type III secretion system (T3SS), and its effectors.

Recently, whole genome sequencing (WGS) strategies towards phytopathogens have provided high-quality sequences to characterize virulence-associated genes [13] and uncovered pathogenic mechanisms for attacking plants [14]. With decreased sequencing costs and rapid development of sequencing techniques, the genomes of diverse bacterial strains have been sequenced. Complete or draft genome analyses have been performed for important phytopathogenic *P. syringae* [15, 16]. The sequencing of multiple strains within a species or pathovar can provide crucial insights into potential evolutionary differences. An in-depth analysis of Psa evolution, based on complete genome sequences of strains isolated from New Zealand, Japan, Korea, and Italy, was conducted to provide evidence for independent transmission events and evolution [17]. Genetic characteristics of Psa biovar 5 and 6 found in Japan provided new findings for the genes involved in host interaction [7, 8]. Comparative genomics helped illustrate the relationships between genotypes and phenotypes [18] and facilitated taxonomic revision [19]. Comparative analysis of Psa isolates from various sources around the world demonstrated their high diversity [8, 20, 21]. Despite the increasing number of research reports on Psa strains, the pathogenic mechanisms and key virulence factors remain unclear.

In this study, we conducted inoculation experiments to investigate the virulence differences of Psa strains that cause bacterial canker in Anhui Province, China. Three representative Psa strains were selected for further analysis. The JZY2 and YXH1 were characterized as mildly virulent strains, while the QSY6 strain was depicted as highly virulent. Whole genome sequencing was carried out to better clarify the detailed characteristics of the three Psa strains employing the Illumina and PacBio systems. Comparison of the three complete genome sequences with 12 Psa strains, the phylogenetic relationships, and ANI analysis revealed that QSY6, JZY2, and YXH1 were closely related to Psa biovar 3 (Psa3) strains. Furthermore, a synteny analysis of the three Psa strains identified unique genomic variations in QSY6. The present study aimed to dissect the genomic profiling of these three Psa strains found in China, assess phylogenetic characteristics, and evaluate the genes responsible for their different virulence capacities involved in invasion and pathogenesis.

## Methods

### Isolates collection and identification of Psa

Three strains named QSY6 (from Qianshan county), JZY2 (from Jinzhai county), and YXH1 (from Yuexi county) used in pathogenicity assays and genome sequencing were originally collected from different areas in Anhui Province, China. QSY6 and JZY2 were isolated from symptomatic leaves of *A. chinensis* var. *chinensis* ‘Donghong’ and ‘Hongyang’, respectively. YXH1 was isolated from symptomatic flowers of *A. chinensis* var. *chinensis* ‘Hongyang’. These three strains were identified by PCR using specific primers for Psa [22] and Psa3 [23], respectively. Then the strains were immediately frozen in glycerol at -80 °C.

### Pathogenicity assays

Pathogenicity tests of the three Psa strains were performed by artificial inoculation of detached canes and leaf discs of *A. chinensis* var. *chinensis* ‘Hongyang’, which were collected from healthy kiwifruit plants in the orchards located in Anhui Province. According to two indoor bioassay methods [21], vacuum infiltration and wound inoculation were conducted for pathogenicity tests. At least 10 canes or leaves were used for each strain in a pathogenicity trial, and repeated for three independent trials. Leaf discs were subjected to vacuum infiltration inoculation with bacteria at a concentration of 10^4^ CFU/mL, while detached kiwifruit canes were inoculated with bacteria at a concentration of 10^8^ CFU/mL through wound. Control treatments were treated with sterile distilled water. The inoculated leaf discs and canes were placed in a climate chamber at 16 h 18 °C:8 h 14 °C, day: night, 90% relative humidity, and the infection symptoms were examined after 5 and 21 days, respectively.

### Genome sequencing, *de novo* assembly and annotation

Genomic DNA extraction, sequence library construction, and sequencing were conducted at Shanghai Biozeron Technology Co., Ltd. Briefly, the concentration and quality of genomic DNA were determined by NanoDrop (ThermoFisher, Loughborough, UK), agarose gel electrophoresis, and TBS-380 fluorometer (Turner BioSystems Inc., Sunnyvale, CA). A highly qualified DNA sample (OD260/280 = 1.8 ∼ 2.0, > 6 ug) was utilized to construct the fragment library. The complete genome sequencing of three strains was performed using the PacBio RS single-molecule real-time (SMRT) platform and Illumina NovaSeq 6000 platform.

The PacBio platform produced approximately 2.4, 3.1, and 1.6 Gb raw reads for QSY6, JZY2, and YXH1, respectively, with an N50 length of 6,892, 6,848, and 6,683 bp. PacBio raw reads were corrected and assembled using Flye v2.8.1 [24]. Illumina short reads were trimmed using Fastp v0.12.4 [25] and then quality checked using FastQC v0.11.9 [26]. The hybrid assembly pipeline was conducted by Unicycler v0.5.0 using both Illumina reads and long reads [27]. Replicate assemblies were clustered, and the most likely contigs were selected based on the evidence of contig circularization, as determined by Unicycler or Flye. Consensus sequences were generated from the selected clusters and polished using NextPolish v1.4.1 [28]. The final circular chromosome and plasmid were manually generated from the resulting sequences fragments. The assembled genome was subsequently annotated using Prokka v1.14 [29], with the reference genomes *P. syringae* pv. *tomato* DC3000 (Pst DC3000). The rRNA and tRNA sequences were predicted and annotated using RNAmmer v1.2 [30] and tRNAscan-SE v1.4 [31], respectively. The genome feature was visualized using CGView Comparison Tool program v2.0.2 [32]. The orthologous groups (COGs), Gene Ontology (GOs), and KEGG were annotated and classified using the EggNog tool v2.0.7 of the BLAST2GO [33].

### Pan-genome analysis

Twelve complete genomes or chromosomes of Psa strains were retrieved from the NCBI genome database (Table S1). All 15 Psa genomes were re-annotated using Prokka v1.14 [29]. The pan-genome analysis of 15 Psa strains was calculated using Roary program v3.13.0 [34] with an identity cutoff of 97%, based on the GFF3 files generated by Prokka [29]. The numbers of core genes and unique genes in the gene presence and absence matrix were presented by R packages (v4.1.2) for the petal plot and UpSet plot.

### Average nucleotide identity and phylogenetic analysis

To estimate the average nucleotide identity among the three strains in this study and 12 reference strains of Psa, whole-genome sequence similarity analysis was performed using bioinformatics tools ANI. The ANI values were calculated by the Python module pyani v 0.2.12 [35] using ANIm parameters to elucidate the intraspecific relationship of these strains. A Pearson correlation matrix was generated and correlation analysis was performed by R packages (v4.1.2).

Single-copy genes were defined as gene clusters that are shared by all strains, with each cluster containing only one gene copy from each strain [36]. The 15 Psa genomes were used to construct a phylogenetic tree. Single-copy orthologous proteins were extracted by OrthoFinder v2.5 [37] with default parameters. The protein sequences obtained from Prokka were selected for each strain and aligned with MUSCLE v3.8 [38]. Then, the best-fit models of amino acid substitution were estimated by ProtTest v3.4 [39], and a ML phylogenetic tree was reconstructed by RAxML v8.2.12 [40]. The Newick tree files were visualized using the online program iTOL v5 [41].

### Type III secretion effectors identification

The type III secretion system effectors (T3Es) were detected as previously described [42]. The presence of T3Es in each strain was identified by BLASTP (E-value < 1 × 10^− 6^) search of the effector proteins in T3Es database of *P. syringae* (http://pseudomonas-syringae.org/). The effector protein was considered present if it matched at least half of the given T3Es, and it was considered absent if no hits were found.

If the alignment was ≥ 25% smaller than the length of the reference T3Es, the putative T3Es were recorded as truncated effectors. *P. syringae* pv. *tomato* DC3000 (Pst DC3000) was used as a reference for the T3SS [43]. A matrix was formulated and employed to construct a dendrogram using the R packages (v4.1.2), based on the presence of complete and incomplete T3Es [44].

### Comparative genomic analyses of three strains

Alignment of the complete genome sequences of the three strains for genomic architectures and syntenic relationships was accomplished with MAUVE v2.4 [45]. The output file “gene_presence_absence.csv” (Table S2), derived from the Roary analysis, was analyzed using Microsoft Excel to determine genes that are exclusive to the QSY6 strain while absent in the other two strains. The genes were screened by the Virulence Factors Database (VFDB) [46], and an E-value cut-off of 1*e*-10 was set for the BLAST analysis.

## Results

### Difference in virulence of QSY6, JZY2, and YXH1 strains

**Fig. 1:**
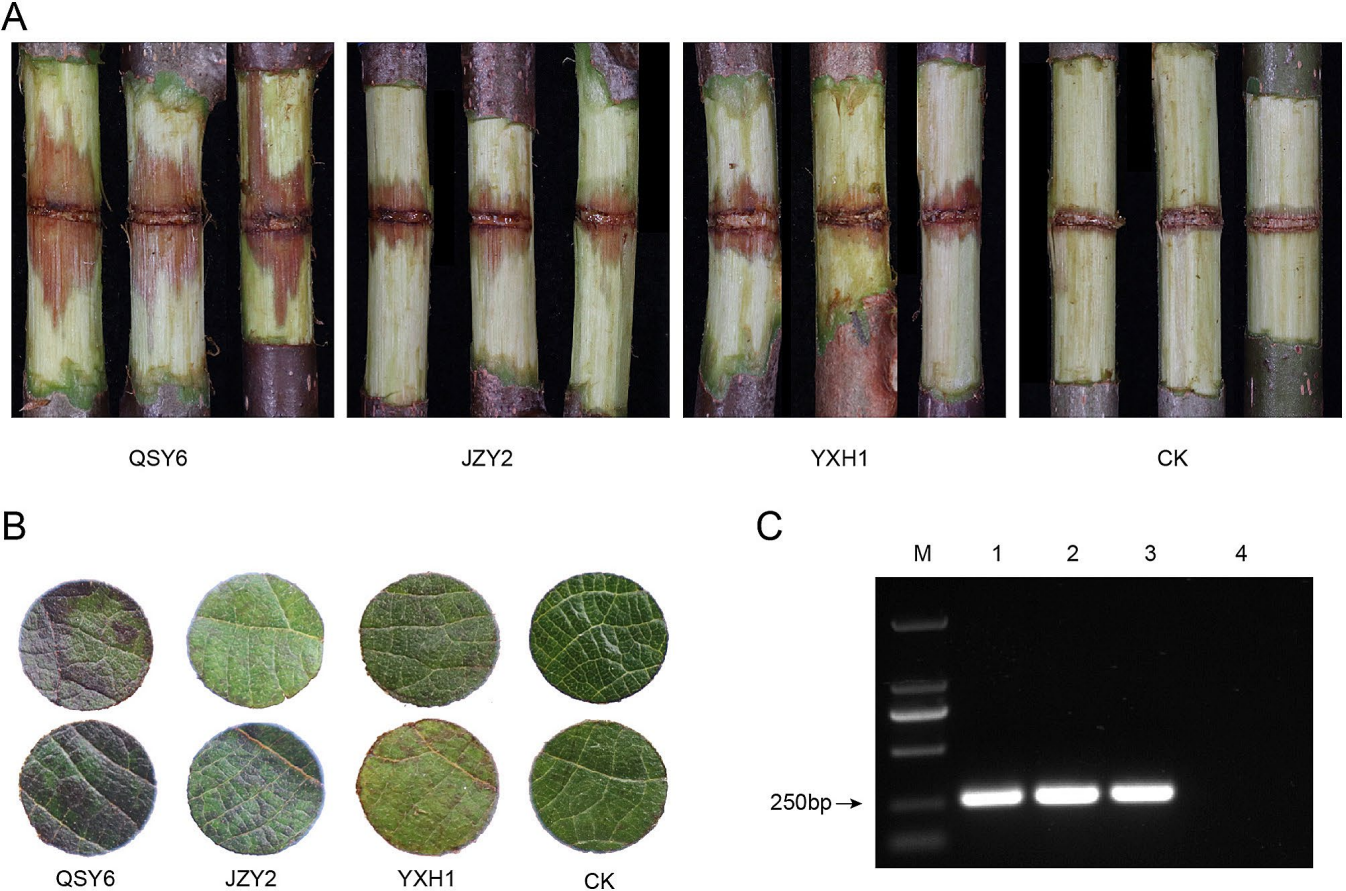
Assessment of the pathogenicity of *Pseudomonas syringae* pv. *actinidiae* (Psa) strains. **(A)** Wound inoculation on the detached kiwifruit canes of *Actinidia chinensis* cv. Hongyang at 21 days post-inoculation (dpi). **(B)** Vacuum infiltration inoculation on leaf discs at 5 dpi. **(C)** PCR identification of Psa biovar 3 (Psa3). Lanes M: Marker; Lanes 1–3: strains QSY6, JZY2, and YXH1. Lane 4: negative control. Figure 1C was cropped and the original gel is presented in Fig. S1

Koch’s postulates were verified by characterizing these strains re-isolated from the symptomatic tissues. Necrotic lesions were observed on the detached canes inoculated with strains QSY6, JZY2, and YXH1. All of these strains were identified as Psa biovar 3 (Psa3), which possessed the target 243 bp DNA fragments (Fig. 1C). The strains were preserved in the China General Microbiological Culture Collection Center as CGMCC 1.62036 (QSY6), CGMCC 1.62037 (JZY2), and CGMCC 1.62078 (YXH1).

Observations made over 21 days showed that the necrotic lesions enlarged. There were no significant differences between strains JZY2 and YXH1, while QSY6 caused significantly larger brown lesions (Fig. 1A). Inoculated leaf discs of *A. chinensis* cv. Hongyang, JZY2, and YXH1 caused necrotic areas 5 days post-inoculation, but it appeared to be less virulent compared to the QSY6 strain (Fig. 1B). It was obvious that QSY6 exhibited stronger pathogenicity when carrying out inoculation experiments, in contrast to JZY2 and YXH1.

### General genomic features of Psa strains QSY6, JZY2 and YXH1

**Fig. 2:**
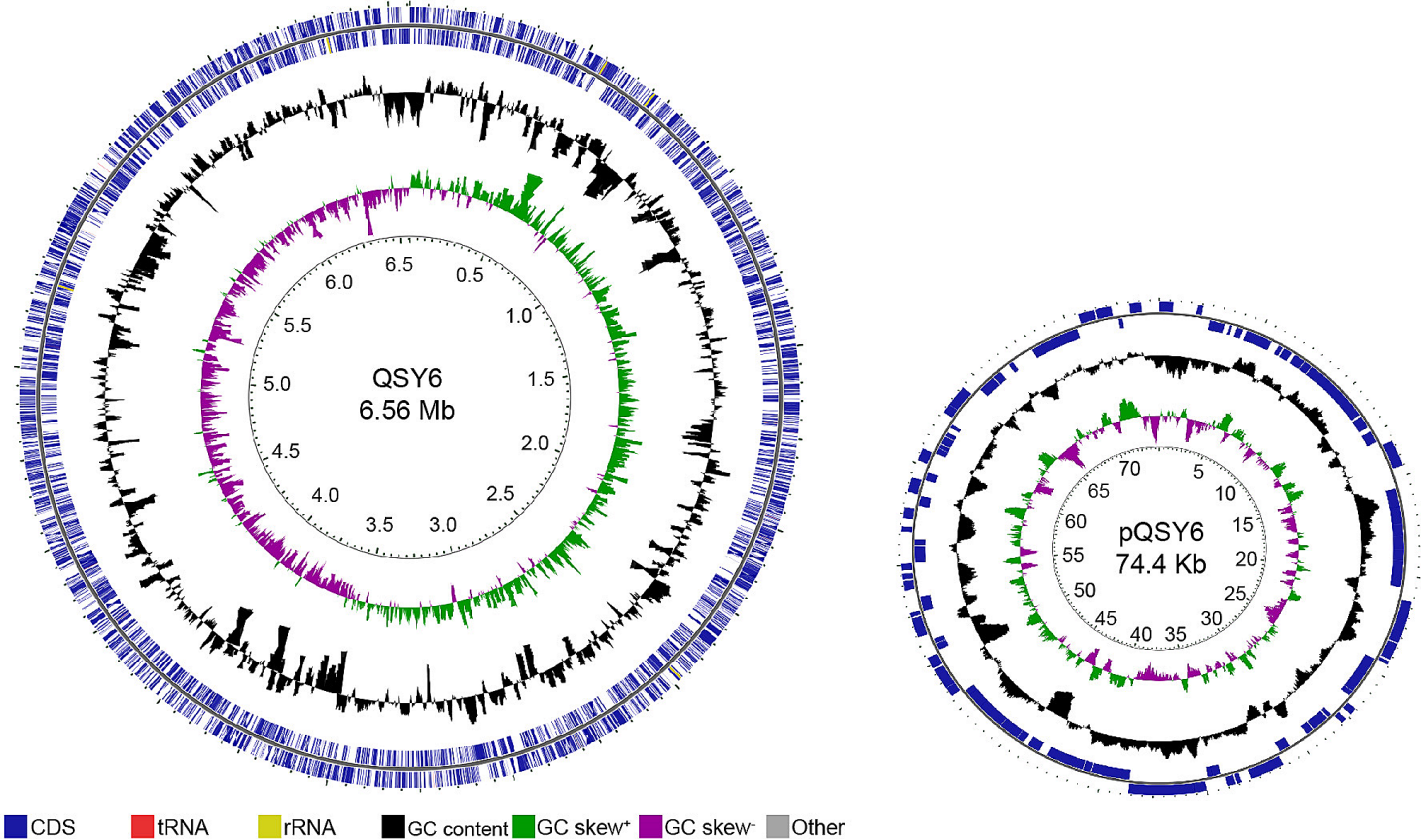
Genome distribution of the *Pseudomonas syringae* pv. *actinidiae* strain QSY6. From the outer to the inner circle: CDS, tRNA, and rRNA on the forward strand; CDS, tRNA, and rRNA on the reverse strand; CDS on the reverse strand colored according to COG category; GC content; GC skew; and genome position in Mbp

To further confirm the taxonomic status and investigate the genomic diversity of Psa strains, three strains (QSY6, JZY2, and YXH1) were *de novo* sequenced based on the PacBio RS and Illumina NovaSeq platforms, with more than 100X coverage depth. The genomes of QSY6, JZY2, and YXH1 consist of a single circular chromosome and a plasmid without gaps, as follows: 6,631,997 (QSY6), 6,502,657 (JZY2) and 6,614,206 bp (YXH1) in size. These genomes contain 6,019, 5,884, and 6,014 protein coding genes, and 68, 66, and 69 tRNAs, respectively. Each genome possesses 16 rRNA and one tmRNA gene (Table 1; Fig. 2, Fig. S2).

To further determine the functional differences due to these protein-coding genes, we analyzed the data using clusters of orthologous groups of proteins (COGs), Gene Ontology (GO), and Kyoto Encyclopedia of Genes and Genomes (KEGG). Our results revealed that 5,160 (85.73%), 5,092 (86.54%), and 5,161 (85.82%) predicted genes of QSY6, JZY2, and YXH1, respectively, were assigned to COG categories (Table S3). Among these assigned genes of the three strains, 36.74–36.31% are related to metabolism, 22.99–22.64% to cellular processes and signaling, and 21.13–21.72% to information. However, 20% of these genes could not be assigned to COG categories because their features and functions are still unknown. As a result, 1,231 (20.45%), 1,227 (20.85%), and 1,229 (20.44%) genes could be assigned with certain GO definitions, respectively. Enriched GO terms focused on cellular process, cellular anatomical entity, obsolete cell part, metabolic process, and organic substance metabolic process (Table S3). Additionally, 745 (12.38%), 742 (12.61%), and 746 (12.40%) genes were mapped and characterized for the functions of proteins according to the KEGG database (Table S3).

**Table 1:** General features of the genomes from the three *Pseudomonas syringae* pv. *actinidiae* strains

Feature	QSY6	JZY2	YXH1
Genome Size	6,631,997	6,502,657	6,614,206
GC content	58.42%	58.46%	58.39%
Contig	2	2	2
Number of coding genes	6,019	5,884	6,014
Number of tRNA	68	66	69
5s rRNA number	6	6	6
16s rRNA number	5	5	5
23s rRNA number	5	5	5
tmRNA	1	1	1

### Pan-core genome analysis

**Fig. 3:**
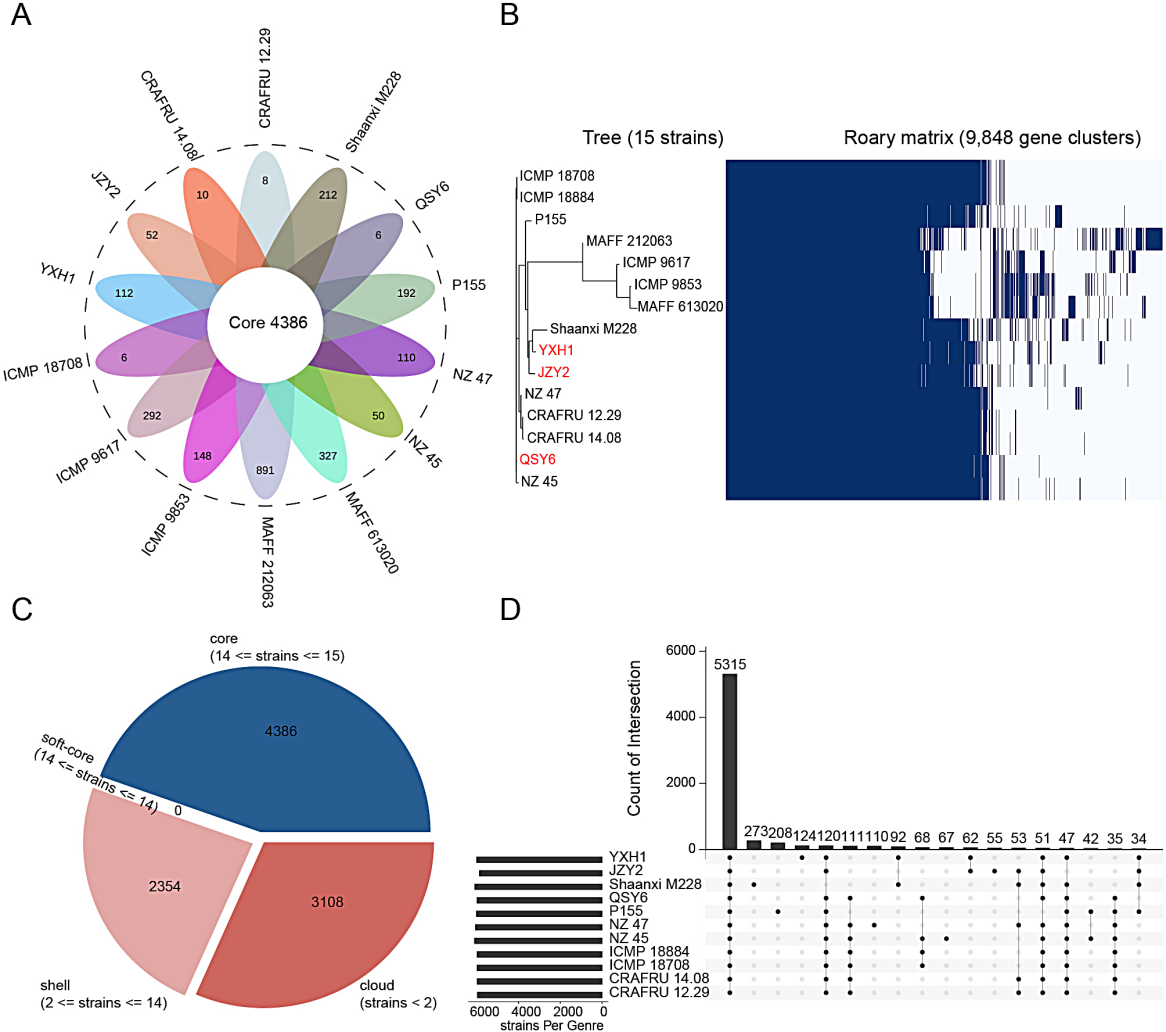
Pangenome analysis of 15 strains of *Pseudomonas syringae* pv. *actinidiae*. **(A)** Flower petal plot of 15 Psa strains. Pan-genome analysis of 15 strains revealed genes unique to each strain, with the corresponding quantities visually represented on individual petal plots. The central Circos diagram illustrates the count of core genes derived from 15 strains. **(B)** Gene presence/absence matrix shows the distribution of genes in each genome. The left panel of the phylogenetic tree illustrates the evolutionary relationships among the strains associated with the corresponding pangenome. The strains from this study are indicated in red color. Dark blue blocks represent the presence of each gene, and white blocks indicate its absence. **(C)** A pie chart represents the number of genes belonging to core, soft-core, shell, and cloud of the Psa strains. **(D)** The UpSet plot showing overlap of orthologous gene clusters across the eleven strains of Psa. Bar numbers are sorted in descending order. Each bar shows the intersection size of core genes with one or more strains. The bar chart on the left panel represents the corresponding strains

To obtain comprehensive results, genome sequences of 12 Psa strains from NCBI database were downloaded and included for pangenome analysis. Highly conserved genes with phylogenetic information constitute the core genes, whereas the flexible part of the genome is comprised of accessory genes (shell and cloud) [47]. Based on 9,848 putative protein-encoding sequences from the genome sequences of these selected strains, 4,386 orthologs were identified as the Psa core genome, comprising 44.54% of the total pangenome, and a high percentage of accessory genes (55.46%) was observed, including 2,354 shell genes and 3,108 cloud genes (Fig. 3A, C). Among them, the frequency of genes with a whole-genome set showed that 2,416 genes were strain-specific in all Psa strains (Fig. 3A). The 15 Psa strains formed four separate clusters on the phylogenetic tree, and the distribution of accessory genes varied among them (Fig. 3B). Characterization of the accessory gene pool could provide information on strain selection and adaptability to the host. The observed extensive accessory genome revealed a high degree of variability among different Psa strains (Fig. 3B), which could be associated with their ability to survive in diverse ecological niches and extensive horizontal gene transfer throughout the strains [48].

According to core genome sequence analysis, JZY2 and YXH1 were closely related and belonged to the same cluster with Shaanxi M228 and P155, which originated from China. In contrast, QSY6 was clustered in a separate clade with NZ 45, which was isolated from New Zealand. In summary, the three sequenced strains were closely related to the eight strains belonging to Psa3, such as Shaanxi M228, P155, ICMP 18884, NZ 45, NZ 47, ICMP 18708, CRAFRU 12.29, and CRAFRU 14.08. However, they were evolutionarily distinct from ICMP 9853 (Psa1), ICMP 9617 (Psa1), MAFF 613020 (Psa1), and MAFF 212063 (Psa5) (Fig. 3B).

Further analysis of the 11 closely related Psa strains discovered 5,315 core genes, 850 unique genes, and 1,105 accessory genes (Fig. 3D, Table S2). Notably, Shaanxi M228 possessed the most abundant unique genes (273), which was much more than other Psa3 strains (Fig. 3D).

### Phylogenomic analysis and average nucleotide identity analysis

**Fig. 4:**
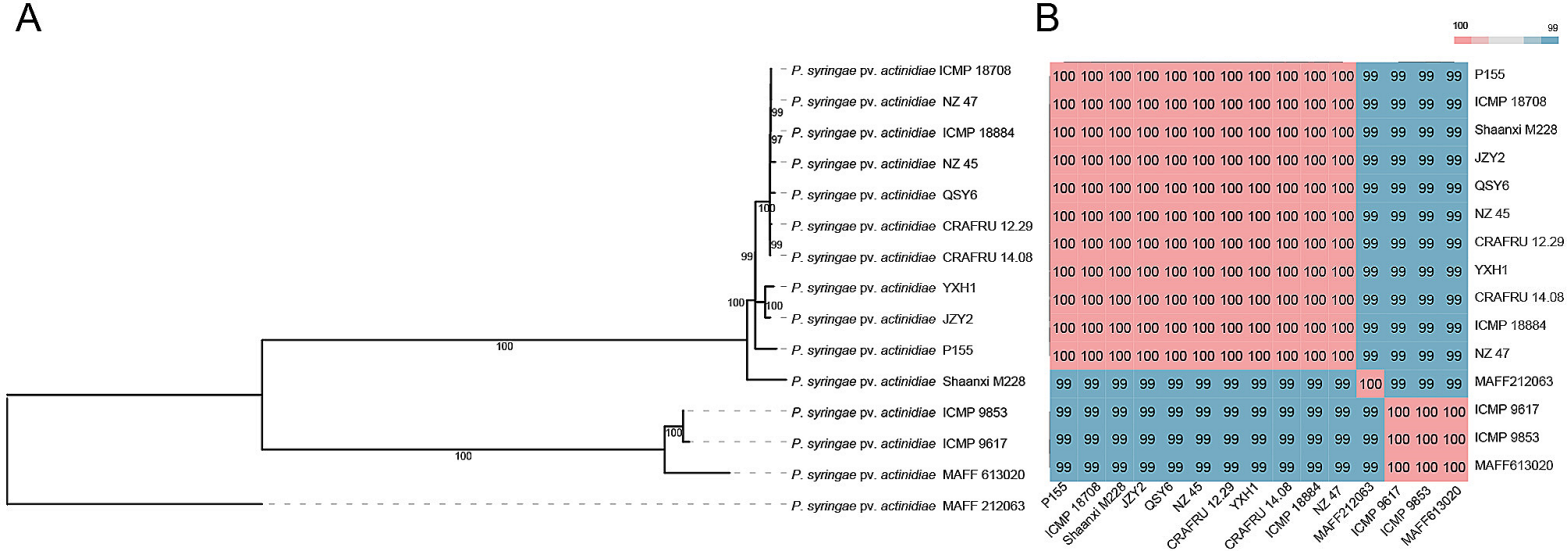
Evolutionary relationships of 15 strains of *Pseudomonas syringae* pv. *actinidiae*. **(A)** Phylogenetic relationship of the sequenced strains QSY6, JZY2, and YXH1 in relation to their closely related genetic relatives. In total, 4,340 single-copy orthologous genes from 15 Psa strains were included in the analysis. **(B)** Heatmap and dendrogram of average nucleotide identity (ANI) values of the 15 Psa strains

To understand the phylogenetic status of the three strains, we constructed a phylogenetic tree and calculated pairwise ANI values for all complete genomes of Psa. The phylogenetic tree, constructed by 4,340 single-copy orthologous groups, showed that three clades were distinguished based on 15 strains from different biovars (Fig. 4A). The phylogenetic analysis, including other Psa strains, showed a clear clustering of different biovars. QSY6, JZY2, and YXH1 were grouped with strains Shaanxi M228 and P155 from China (Sichuan Province and Shaanxi Province), strains ICMP 18884, NZ 45, NZ 47, and ICMP 18708 from New Zealand, strain CRAFRU 12.29 from Italy, and strain CRAFRU 14.08 from Portugal, all designated as Psa3. In addition, strains ICMP 9853, ICMP 9617, and MAFF 613020 were classified as biovar 1 (Psa1) from Japan and formed a monophyletic group, distant from strain MAFF 212063 (identified from Japan as biovar 5). The phylogenetic relationships revealed that JZY2 and YXH1 (lowly virulent) were more closely related to strains (P155, Shaanxi M228) from China, while QSY6 (highly virulent) was tightly clustered with Psa3 from New Zealand, Italy, and Portugal (Fig. 4A).

The 15 strains of Psa grouped in the same phylogenetic clade also showed a high ANI value with each other. Regarding the threshold, three different clades with a similarity level of at least 99% were observed within the strains (Fig. 4B). The three strains grouped (QSY6, JZY2, and YXH1) in clade 1 with the other eight Psa strains were classified as Psa3. Strains ICMP 9853, ICMP 9617, MAFF 613020 (Psa1) have a lower ANI similarity (99%) than MAFF 212063 (Psa5) and other members, differing from members of Psa3, justifying classification into separate phylogenetic groups. The results of ANI showed a similar topology to that present in the phylogenetic tree.

### Effector distribution in Psa strains

**Fig. 5:**
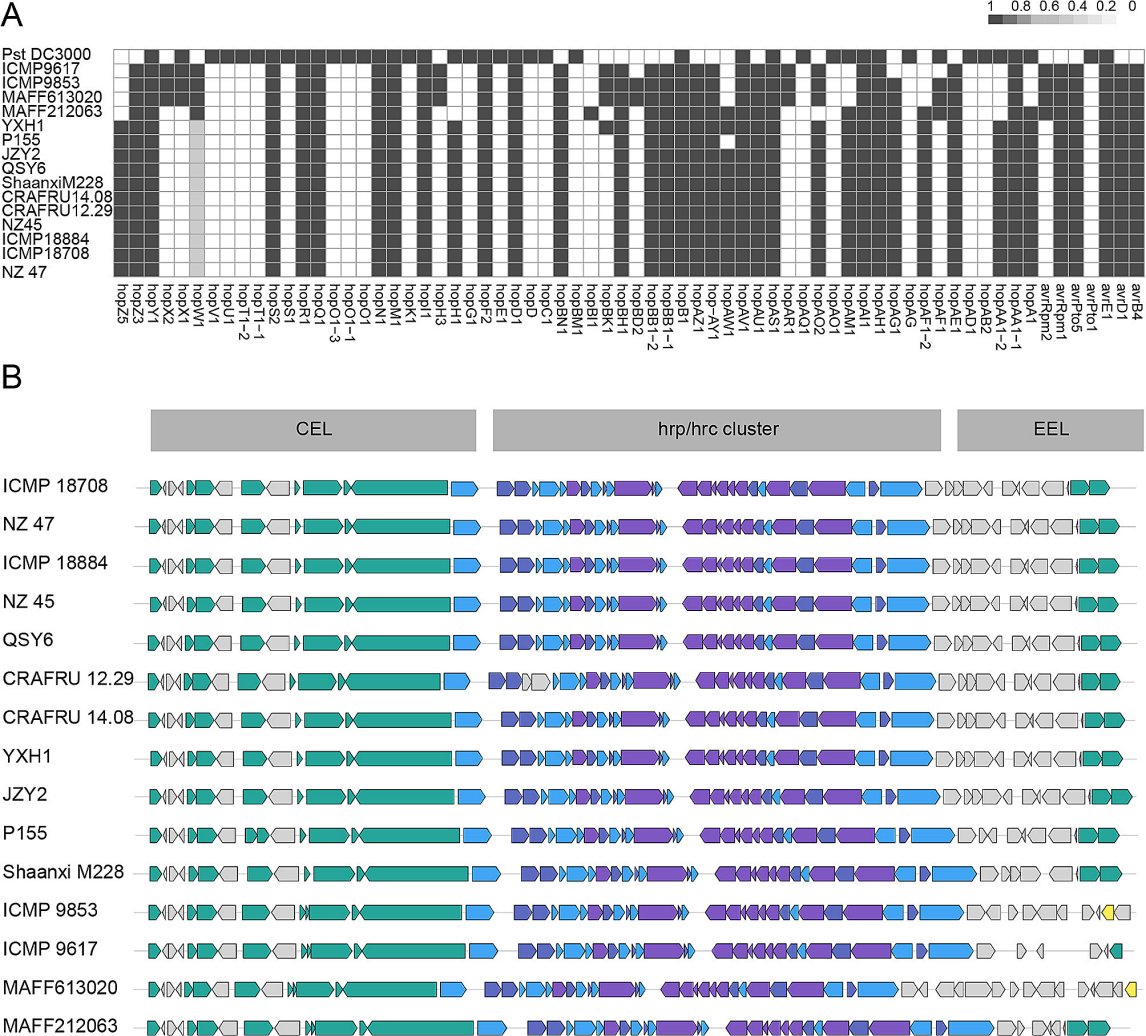
Type III secretion system (T3SS) effector repertoires and organization. **(A)** The phylogenetic patterns of effector families presence/absence in the 15 *Pseudomonas syringae* pv. *actinidiae* (Psa) strains and *P. syringae* pv. *tomato* DC3000 effector repertoires. Gene presence is illustrated with colored cells in the heatmap, the presence of full-length protein is colored in black boxes, incomplete alignments (characterized by an alignment length < 25% of the reported T3SS length) are represented by grey boxes, and the absence of significant matches is indicated by white boxes. **(B)** The structural organization of the T3SS gene clusters of the 15 Psa strains and Pst DC3000, including *hrp*/*hrc* genes, conserved effector locus (CEL), and exchangeable effector locus (EELs)

Translocating virulence factors via the type III secretion system to the host cell is a strategy employed by most pathogenic Gram-negative bacteria during the infection process, suggesting most of the type III secreted effectors (T3SEs) predicted in Psa contribute to virulence [49]. In this study, a representation of effector repertoire composition was generated spanning the diversity among 15 strains of Psa and the strain Pst DC3000 (Fig. 5A). The highest number of T3SS effectors (42 effectors) was detected in the genome of the strain Pst DC3000 (Fig. 5A), which is consistent with a previous study [50]. In general, each strain of Psa contained more than 38 effector genes. We were able to reveal the repertoire of more than 38 effectors in all 15 Psa strains. Pst DC3000 and the 15 Psa strains shared only 16 effector genes, while 30 core effectors were presented among the 15 Psa strains (Fig. 5A).

Distribution of effector genes among phylogenetically diverse strains revealed a set of core and variable effectors, suggesting that several effectors have been acquired and lost in diverse biovars of Psa. The effector repertoire was similar among the entire Psa3, but differed from Psa1. A notable feature of Psa effector repertoires is that divergent repertoires could be found in biovar 1 (Fig. 5A). Of all the Psa strains compared, the effectors hopX1, hopBK1, hopBD2, avrRpm2, hopX2, hopAR1, hopH3, and hopAF1 were detected only in Psa1, while hopH1, hopAA1-2, hopAM1, hopA1, hopZ5, and hopAF1-2 were detected only in Psa3. The T3SS effector hopBK1 was exclusively detected in the genome of YXH1, while it was not present in the genomes of strains QSY6 and JZY2. Notably, and in agreement with the phylogenetic analysis shown in Fig. 4A, the strains close to Psa3 contained the same T3Es repertoire as the three sequenced strains.

The comparison between the *hrp-hrc* gene cluster that encodes structural components and regulators of the T3SS showed that Psa strains possess the conserved effector locus (CEL) and the exchangeable effector locus (EEL) (Fig. 5B). The gene cluster of Psa3 strains was identical with each other, while the EEL of Psa1 strains ICMP 9853, ICMP 9617, MAFF 613020, and Psa5 strain MAFF 212063 was different from those of Psa3.

### Virulence factors contribute to high-virulence of QSY6

**Fig. 6:**
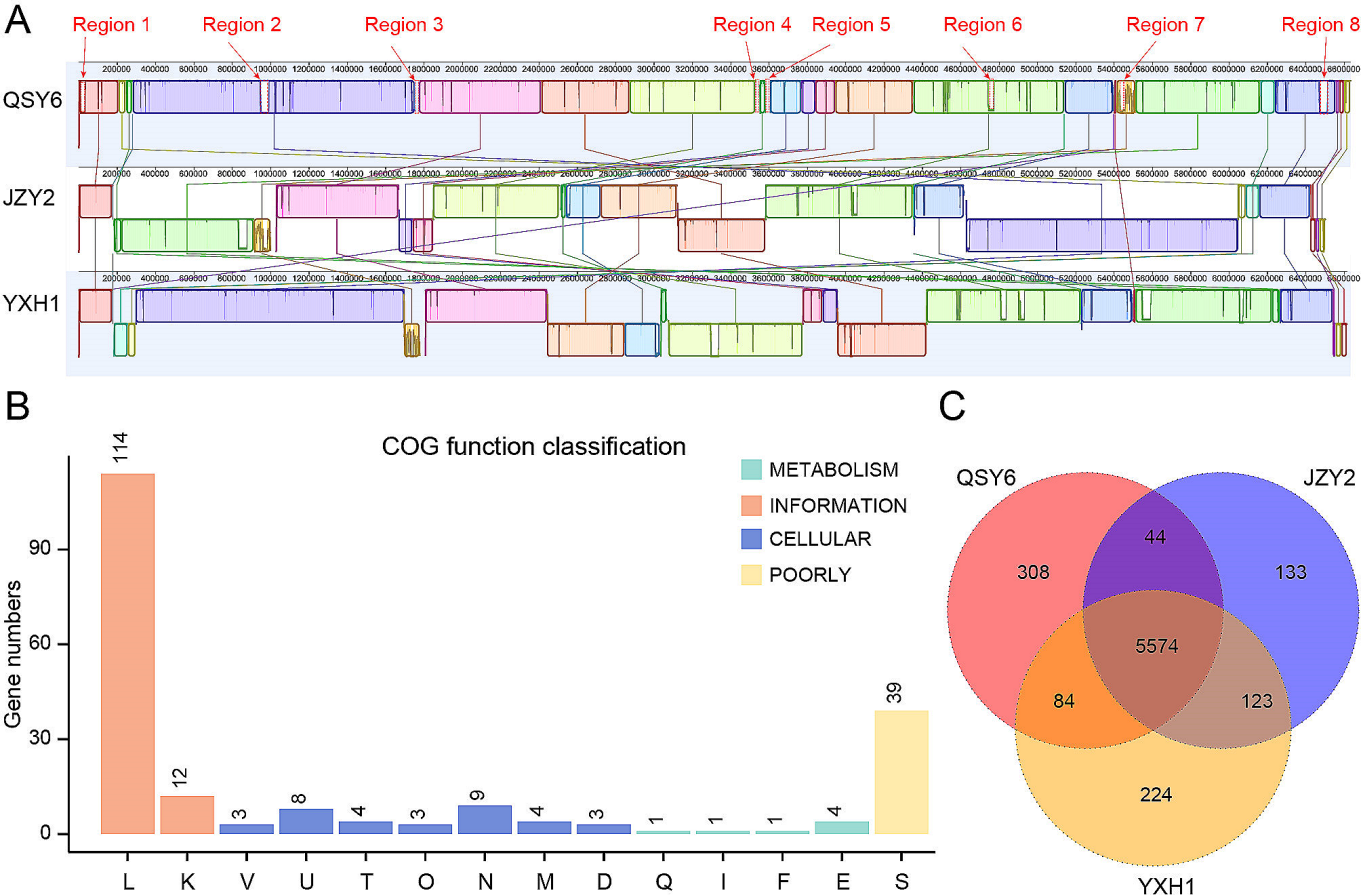
Complete genome alignment of three strains of *Pseudomonas syringae* pv. *actinidiae* (Psa). **(A)** Syntenic alignment among three strains of *P. syringae* pv. *actinidiae* strains (QSY6, JZY2, and YXH1), demonstrating the close overall similarity between each strain chromosome, and showing the different regions of QSY6. Corresponding colored boxes represent locally collinear blocks (LCBs) or homologous DNA regions shared among genomes. Uncolored regions within the genomes indicate loci harboring strain-specific sequences. **(B)** COG function classification of unique genes present in QSY6, grouped into four main parts: Metabolism, cellular processes, information, and poorly. **(C)** Venn diagram illustrates the number of unique and core genes in the three strains. The numbers of unique genes for each strain are shown on each plot, as well as the center circle shows the number of core genes common to the three strains

The high-virulence strain QSY6 was compared with the low-virulence strains JZY2 and YXH1 to investigate the collinear relationship and orthologous distribution of genes. As a result, a high degree of synteny was demonstrated among the three strains (regions with the same color). While a total of 8 distinctive genomic regions were detected in QSY6 (Fig. 6A), which were obviously different from JZY2 and YXH1. More than 85% of gene clusters that were core to the three genomes, and we identified 308 unique genes corresponding to distinctive genomic regions in QSY6 (Fig. 6C, Table S4-S5). Most of these genes have been categorized within the information processing category of COG annotations (Fig. 6B).

In the case of pathogenic bacteria, strain-specific genes frequently encode important virulence factors [51]. Herein, based on the VFDB database [46], we found that 16 out of 308 virulence genes in the QSY6 genome are quite different from those of JZY2 and YXH1 after BLASTP searching (Table S5, Table 2). Of these, five genes QSY6_05046, QSY6_05047, QSY6_05065, QSY6_05082, QSY6_05216 encoding for the VFDB factors rvhB, aec7, acfB, pilT, and HopAC1, respectively (Table S5, Table 2). These VFDB factors were assigned to Effector delivery systems, responsible for transporting secretory proteins and effector proteins. As described previously, the type III secretion system effector E3 ligases ipaH2.5 plays an important role in *Shigella flexneri* [52]. HopAC1 contributes to the virulence of Psa, either partially or during the epiphytic phase at leaf surfaces [53]. Type IV pili have been shown to be essential for host colonization, adherence, biofilm formation, and their role as important determinants of pathogenicity has been established in phytopathogens [54]. Our results showed that 11 genes were annotated to Adherence, which are involved in the synthesis of type IV pilus components and related to bacterial biofilm formation. These results suggested that the candidate genes located in distinctive genomic regions might be responsible for the high virulence of QSY6.

**Table 2:** The annotation of 16 specific-genes in QSY6 using the VFDB databases

Gene ID	Gene Annotation	VFDB	VFDB categories
QSY6_01593	ISPsy12, transposase OrfB	ipaH2.5	Effector delivery systems
QSY6_03284	ISPsy12, transposase OrfB	ipaH2.5	Effector delivery systems
QSY6_04997	conj_PilL	pilL	Adherence
QSY6_04998	pilus_B_mal_scr	pilN	Adherence
QSY6_04999	hypothetical protein	pilO	Adherence
QSY6_05001	type_IV_pilB	pilQ	Adherence
QSY6_05002	Toxin coregulated pilus biosynthesis protein E	pilR	Adherence
QSY6_05003	hypothetical protein	pilS	Adherence
QSY6_05004	Twitching mobility protein	pilU	Adherence
QSY6_05005	hypothetical protein	pilV	Adherence
QSY6_05010	methyl-accepting chemotaxis protein	acfB	Effector delivery systems
QSY6_05046	hypothetical protein	rvhB1	Adherence
QSY6_05047	ICE_PFL_4695	aec7	Adherence
QSY6_05065	methyl-accepting chemotaxis protein	acfB	Effector delivery systems
QSY6_05082	conj_TIGR03755	pilT	Adherence
QSY6_05216	hypothetical protein	hopAC1	Effector delivery systems

## Discussion

Significant variations in virulence, genotypes, and geographical distribution among Psa biovars are being observed. Since 2008, Psa3 has emerged as the most severe virulent group, leading to global pandemics [8, 55]. The Psa strains isolated from China had previously been assigned to the biovar 3 group [21, 56]. In this study, the molecular identification and genomic population analyses confirmed that the three sequenced Psa strains (QSY6, JZY2, and YXH1) isolated from Anhui Province of China belong to biovar 3. It was supposed that Psa3 might be a prevalent species that causes canker to the local kiwifruits.

The genetic analysis based on whole genomes instead of a few housekeeping genes has provided more reliable evidence in classification and evolution [47, 57]. The comparison of all available genomic information could distinguish biovars clearly. Phylogenetic studies based on single-copy orthologous groups have made a significant contribution to classification of bacteria [58]. The phylogenetic results demonstrated that three sequenced strains were grouped together with biovar 3 strains isolated from China [59, 60], New Zealand [55, 61], Italy [55], and Portugal [62], but far away from Japan [6, 7]. ANI analysis and pangenome analysis confirmed the grouping pattern of biovars, which was consistent with the phylogenetic relationship previously described [10, 63]. Pathogenicity tests conducted on the detached kiwifruit canes revealed variations in virulence among the three strains obtained from different kiwifruit orchards. The pathogenic strains of Psa harbors a diverse weaponry of virulence factors, including the type III secretion system (T3SS) and its effectors, phytotoxins, and flagella. The deletion of individual T3SS effectors in strains typically results in a relatively subtle impact on pathogenesis [64, 65]. Considering different biovars, the variations were observed in the organization of the type III secretion system (T3SS) and the repertoire of effector proteins. Previous studies have shown that the effector hopA1 is specifically present in Psa3 strains but absent in Psa1 [9, 66]. The expression of HopAF1-2 was found to significantly enhance the competitiveness of *P. savastanoi* pv. *savastanoi* strain NCPPB 3335 in *Nicotiana benthamiana* [67]. Furthermore, the function of HopAF1-2 proved adequate to overcome plant defense and partially restore the growth ability of Pst DC3000 in plants [68]. In this study, HopAF1-2 was found exclusively in Psa3, suggesting that it may contribute to the high virulence of Psa3. Previous studies have reported that the effector HopZ5 was among several T3SEs present exclusively in the woody hosts pathogens [69], and was confirmed that HopZ5 was also one of the few virulent factors associated with virulence of Psa3 strains [21, 66]. Furthermore, the *in-frame* deletion of the hopZ5-hopH1 cluster in Psa strain M228 led to a severely reduced pathogenicity phenotype [21]. Here, we confirmed that effectors hopA1, hopAF1-2, hopZ5, and hopH1 were detected only in Psa3.

Genome-wide comparisons of microorganisms need to be comprehensively systematized, based on conserved and variable regions in the genomes, determination of expression profiles, and the correlation of phenotypic characteristics [70]. A Whole-genome comparison of the three sequenced genomes further revealed variable regions and distinct genes associated with highly virulent strains. The high-virulence strain QSY6 displayed distinctive genomic regions corresponding to a significant portion of distinct genes. The specific genes have been categorized within the information processing category of COG annotations, as well as annotated as Effector delivery systems and Adherence of VFDB categories. The different virulence-associated gene repertoires of these three strains might be responsible for the evident difference in pathogenicity detected *in planta*. Pathogenic bacteria deliver virulence factors such as effectors into host cells in order to facilitate infection [52]. As adherence plays a crucial role in the development of biofilms and tissue invasion, the binding capacity is closely associated with their pathogenicity [71]. QSY6 exhibits enhanced virulence in terms of adherence to and invasion of plants, potentially attributed to the specificity of Type IV pili.

In this study, comparative analysis of the complete genomes of three Psa strains, along with 12 Psa reference strains, unveiled genomic features and biovars classification. The distinct pathogenicity of the three strains was evaluated, potentially associated with virulence factors involved in effector delivery and biofilm formation during invasion. Furthermore, the T3SS effector analysis provided valuable data for confirming the effector repertoire of novel T3SS effectors and the distribution of structural components, suggesting that these candidate genes encoding specific effectors should be investigated in further research.

## Conclusions

In summary, PCR-specific detection assays and comparative genomic analysis of three Psa strains isolated from Anhui Province, China, confirmed them as Psa biovar 3. The analysis of ANI, phylogenetic relationships, and T3SEs distribution among three sequenced Psa strains and other 12 representative Psa strains exhibited distinct clustering patterns corresponding to different biovars. Notably, pathogenicity assays indicated that strain QSY6 exhibited higher virulence than the other two strains. Genome synteny analysis of these three strains showed that QSY6-specific regions contained virulence-related genes associated with effector delivery systems and adherence, which may be necessary for the high virulence of strain QSY6. This work provided genomic resources for three Psa strains and preliminarily investigated their differences in virulence, contributing to a deeper understanding of the pathogenesis of Psa.

### Electronic supplementary material

Below is the link to the electronic supplementary material.

Supplementary Material 1

Supplementary Material 2

Supplementary Material 3

Supplementary Material 4

Supplementary Material 5

Supplementary Material 6

Supplementary Material 7

## Data Availability

The original contributions presented in the study are publicly available. All sequencing data of *P. syringae* pv. *actinidiae* QSY6, JZY2, and YXH1 have been deposited in the GenBank database under the accession Bioproject ID PRJNA1013226, PRJNA1022518, and PRJNA1025040, respectively. All three assembled genomes and annotations were deposited at https://zenodo.org/records/10983855.
