# Supplementary material for: Comparative genomic analyses provide insight into the pathogenicity of three Pseudomonas syringae pv. actinidiae strains from Anhui Province, China
Source: BMC Genomics. 2024 May 11;25:461. doi: 10.1186/s12864-024-10384-1 (PMC11088785; doi:10.1186/s12864-024-10384-1)
Supplement: Supplementary file 1 — Supplementary Material 1 [file 12864_2024_10384_MOESM1_ESM.docx]

**Tables**

Table S1. Characteristics of strains used in this study.

Table S2. Genes presence and absence in 15 strains of *Pseudomonas syringae* pv. *actinidiae*. The presence of each CDS in the respective genome is listed below each labeled strain column. If a cell is blank the respective strain does not have the gene.

Table S3. Gene annotation by COG, GO, and KEGG for *Pseudomonas syringae* pv. *actinidiae* (Psa) strains QSY6, JZY2, and YXH1.

Table S4. Genes presence and absence in three *Pseudomonas syringae* pv. *actinidiae* (Psa) strains. The presence of each CDS in the respective genome is listed below each labeled strain column. If a cell is blank the respective strain does not have the gene.

Table S5. Unique genes identification and annotation in QSY6, compared with JZY2, and YXH1.
