# Supplementary material for: Comparative genomic analyses provide insight into the pathogenicity of three Pseudomonas syringae pv. actinidiae strains from Anhui Province, China
Source: BMC Genomics. 2024 May 11;25:461. doi: 10.1186/s12864-024-10384-1 (PMC11088785; doi:10.1186/s12864-024-10384-1)
Supplement: Supplementary file 7 — Supplementary Material 7 [file 12864_2024_10384_MOESM7_ESM.docx]

**Supplementary Information**

**Figures**

Fig. S1. The original, uncropped DNA Electrophoretic in the paper.

Fig. S2. Genome distribution of *Pseudomonas syringae* pv. *actinidiae* (Psa) strains. (A) Genome distribution of Psa strain JZY2 (B) Genome distribution of Psa strain YXH1.

**Fig. S1**


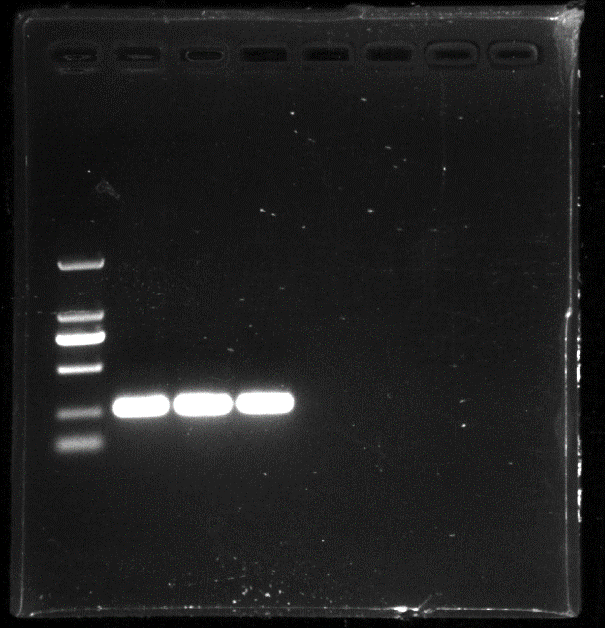
A


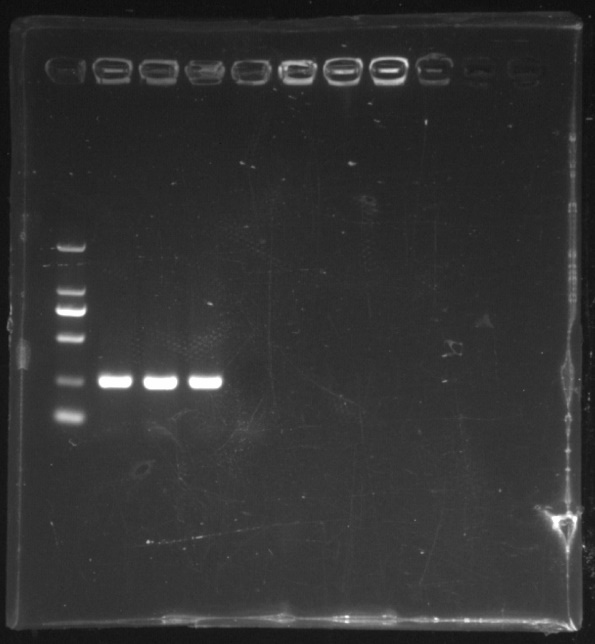
B

Fig. S1. A The original, uncropped DNA Electrophoretic in the paper Fig.1C. B Repeated experiment, the original, uncropped DNA Electrophoretic. The lanes from left to right are Marker, QSY6, JZY2, YXH1, and negative control.

**Fig. S2**


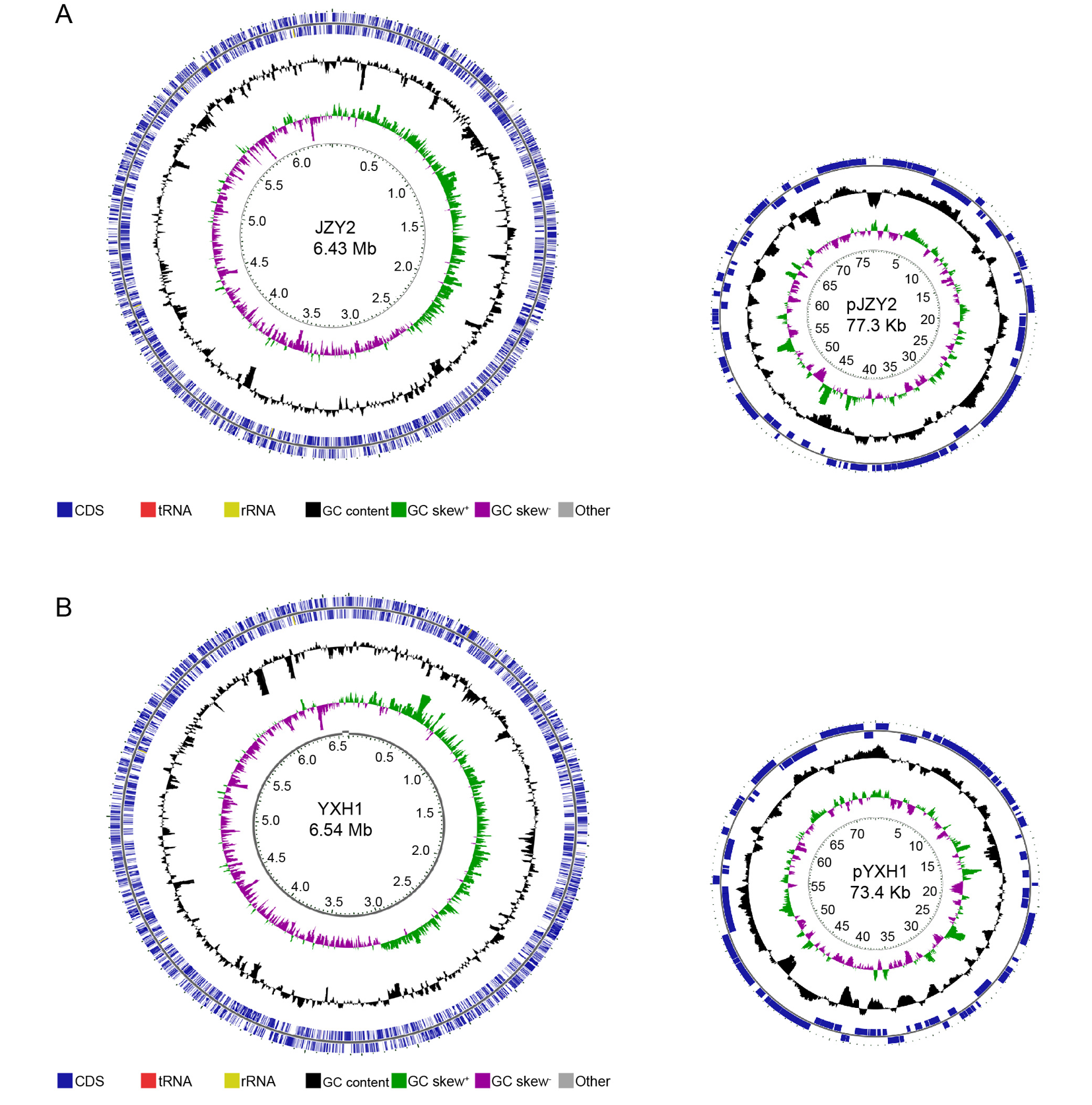


Fig. S2. Genome distribution of *Pseudomonas syringae* pv. *actinidiae* (Psa) strains. (A) Genome distribution of Psa strain JZY2 (B) Genome distribution of Psa strain YXH1. From the outer to the inner circle: CDS, tRNA, and rRNA on the forward strand; CDS, tRNA, and rRNA on the reverse strand; CDS on the reverse strand colored according to COG category; GC content; GC skew; and genome position in Mbp.
